# The combination of gefitinib and RAD001 inhibits growth of HER2 overexpressing breast cancer cells and tumors irrespective of trastuzumab sensitivity

**DOI:** 10.1186/1471-2407-11-420

**Published:** 2011-10-01

**Authors:** Wieslawa H Dragowska, Sherry A Weppler, Mohammed A Qadir, Ling Yan Wong, Yannick Franssen, Jennifer HE Baker, Anita I Kapanen, Guido JJ Kierkels, Dana Masin, Andrew I Minchinton, Karen A Gelmon, Marcel B Bally

**Affiliations:** 1 Experimental Therapeutics, British Columbia Cancer Agency, 675 West 10th Ave, Vancouver, BC, V5Z 1L3, Canada; 2 Integrative Oncology, British Columbia Cancer Research Centre, Vancouver, BC, Canada; 3 Faculty of Pathology and Laboratory Medicine, University of British Columbia, Vancouver, BC, Canada; 4 Medical Oncology, British Columbia Cancer Agency, Vancouver, BC, Canada; 5 Faculty of Pharmaceutical Sciences, University of British Columbia, Vancouver, BC, Canada; 6 Center for Drug Research and Development; Vancouver, BC, Canada

## Abstract

**Background:**

HER2-positive breast cancers exhibit high rates of innate and acquired resistance to trastuzumab (TZ), a HER2-directed antibody used as a first line treatment for this disease. TZ resistance may in part be mediated by frequent co-expression of EGFR and by sustained activation of the mammalian target of rapamycin (mTOR) pathway. Here, we assessed feasibility of combining the EGFR inhibitor gefitinib and the mTOR inhibitor everolimus (RAD001) for treating HER2 overexpressing breast cancers with different sensitivity to TZ.

**Methods:**

The gefitinib and RAD001 combination was broadly evaluated in TZ sensitive (SKBR3 and MCF7-HER2) and TZ resistant (JIMT-1) breast cancer models. The effects on cell growth were measured in cell based assays using the fixed molar ratio design and the median effect principle. *In vivo *studies were performed in Rag2M mice bearing established tumors. Analysis of cell cycle, changes in targeted signaling pathways and tumor characteristics were conducted to assess gefitinib and RAD001 interactions.

**Results:**

The gefitinib and RAD001 combination inhibited cell growth *in vitro *in a synergistic fashion as defined by the Chou and Talalay median effect principle and increased tumor xenograft growth delay. The improvement in therapeutic efficacy by the combination was associated *in vitro *with cell line dependent increases in cytotoxicity and cytostasis while treatment *in vivo *promoted cytostasis. The most striking and consistent therapeutic effect of the combination was increased inhibition of the mTOR pathway (*in vitro *and *in vivo*) and EGFR signaling *in vivo *relative to the single drugs.

**Conclusions:**

The gefitinib and RAD001 combination provides effective control over growth of HER2 overexpressing cells and tumors irrespective of the TZ sensitivity status.

## Background

HER2 overexpression is present in 13-30% of all breast cancers [1,2] and it correlates with poor disease outcome, high rates of metastasis and resistance to conventional treatment modalities [1-5]. Trastuzumab (TZ; Herceptin^®^), a monoclonal antibody that targets the HER2 receptor and interferes with its function is effective in treating some HER2-positive breast cancers [6-8]. However, many patients with HER2-positive disease are insensitive to TZ both as first line treatment or following a relapse after conventional chemotherapy [6-9]. Furthermore, the majority of patients with metastatic disease that initially respond to TZ ultimately develop clinically relevant resistance to this agent [8,9]. As TZ treatment has recently been expanded into the adjuvant setting [10], intrinsic and acquired resistance represents an important clinical problem that urgently awaits a discovery of novel drugs and development of innovative drug combinations to improve outcome for patients with advanced HER2-positive and TZ refractory disease.

Numerous studies have demonstrated that HER2 is often co-expressed in breast cancers with epidermal growth factor receptor (EGFR) [1,5,8,11-16]. It has been established that dimerization of HER2 and EGFR generates a potent signaling response mediated primarily through activation of the phosphatidylinositol 3-kinase (PI3K)/AKT and the RAS-Raf-mitogen-activated protein kinase (MAPK) pathways that sustain cancer cell growth, proliferation and survival [5,8]. Co-expression of EGFR and HER2 in breast cancer cell lines has been shown to induce drug resistance, including resistance to TZ [17,18], and has been correlated with a negative prognosis for breast cancer patients [1,11]. These data suggested that EGFR constitutes an important therapeutic target in breast cancers and have prompted investigators to consider gefitinib (ZD1839, Iressa^®^), a reversible small molecule inhibitor of the EGFR tyrosine kinase, for treatment of HER2 overexpressing and EGFR co-expressing breast malignancies [19].

The preclinical data have demonstrated that gefitinib exerts positive therapeutic effects in models of HER2 overexpressing breast cancer which have been attributed to blocking activity of the PI3K/AKT and the MAPK pathways, increased apoptosis, induction of cytostasis through G_1_/G_0 _cell cycle arrest and downregulation of cyclin D1, as well as inhibiting angiogenesis [12-14,20,21]. However, our previous study conducted in animals bearing HER2 overexpressing MCF7-HER2 and MDA-MB-435/LCC6-HER2 breast cancer xenografts showed that gefitinib monotherapy results in only modest reduction of tumor volume [12]. The same study also showed that when gefitinib was used in combination with TZ the *in vivo *efficacy has been improved as judged by inhibition of tumor growth, but the data obtained by measuring multiple endpoints of therapeutic activity revealed that the combination was not beneficial [12]. These results have been recapitulated in a clinical trial demonstrating that the TZ and gefitinib combination should not be used for treatment in patients with HER2-positive breast cancer [19].

More recently, it has been shown that HER2 overexpression in breast cancer is often associated with aberrant activation of the mTOR pathway [22,23]. mTOR is a major cellular signaling hub that integrates inputs from the upstream signaling pathways, including tyrosine kinase receptors, while also governing energy homeostasis and cellular responses to stress such as nutrient deprivation and hypoxia [24,25]. The mTOR kinase liaisons with either Raptor or Rictor proteins to form two functionally different complexes: rapamycin-sensitive mTOR complex 1 (mTORC1) and rapamycin-insensitive mTOR complex 2 (mTORC2) [24,25]. The most prominent downstream effectors of mTORC1 include ribosomal S6 kinase (S6K) and the eukaryotic translation initiation factor 4E-binding protein 1 (4E-BP1) which regulate the translation of ribosomal and cap-dependent proteins essential for cell growth and G_1 _to S cell cycle progression [24,25]. mTORC2 is an Akt Ser473 kinase that is controlled by a feedback inhibitory loop mediated through S6K1 (p70S6K) [24-29]. Because of its critical role in promoting cell growth, mTOR is considered an attractive target in cancer [25,30]. Everolimus (RAD001) and CCI-779 are two allosteric mTORC1 inhibitors that are in clinical development for various malignancies; however, single-agent therapy has only modest efficacy in the metastatic breast cancer setting [31,32]. These results have encouraged the investigation of mTORC1 inhibitors in combination with other targeted therapies such as aromatase inhibitors and HER2 targeting drugs. A Phase I/II trial of RAD001 in combination with TZ in refractory HER2 positive metastatic breast cancer have reported encouraging results with 34% of patients achieving clinical benefit [33]. Interestingly, several preclinical studies documented that mTOR inhibitors combined with EGFR targeted agents increase efficacy of treatment in renal, lung, pancreatic, colon, prostate and HER2-negative breast cancer models [34-36]. However, the therapeutic effects of EGFR and mTOR inhibitors in combination have not yet been broadly assessed in HER2 overexpressing breast cancers with different TZ sensitivity. Here, we show that the EGFR inhibitor gefitinib and the mTOR inhibitor RAD001 when used in combination improve effectiveness of the treatment in HER2 overexpressing breast cancers that results in impediment of cancer growth.

## Methods

### Cells, tumor xenografts and treatments

MCF7-HER2 cells were a gift from Dr. M. Alaoui-Jamali (McGill University, Montreal, Quebec, Canada) [37], SKBR3 cells were purchased from American Type Culture Collection (ATCC) and JIMT-1 cells [38] were purchased from German Collection of Microorganisms and Cell Culture (Deutsche Sammlung von Mikroorganismen und Zellkulturen GmbH (DSMZ). All cell lines were tested Mycoplasma negative by PCR reaction. MCF7-HER2 cells were maintained in RPMI, SKBR-3 in McCoy's 5A and JIMT-1 in DMEM supplemented with L-glutamine and 10% fetal bovine serum. For *in vivo *studies JIMT-1 and MCF7-HER2 cells were harvested in the exponential growth phase and 5 × 10^6 ^(JIMT-1) or 1 × 10^7 ^(MCF7-HER2) cells were injected subcutaneously (s.c.) on the back of female Rag2M immuno-compromised mice. Mice receiving MCF7-HER2 cells were implanted with 17-β-estradiol 60-day release tablets (IRA, Sarasota, FL) one day prior to tumor inoculation. Tumor growth was monitored twice a week; tumor sizes were calculated using the formula: 0.5 [length (mm)] × [width (mm)^2^]. All agents were delivered as oral gavage. Treatment was initiated on day 17 and carried-out Monday through Friday (QDx5) for 28 (JIMT-1) or 25 (MCF7-HER2) days. RAD001 (a generous gift from Novartis) was diluted with vehicle (Novartis, content not disclosed) and aliquots were kept frozen for the course of treatment. RAD001 and vehicle aliquots were thawed 10-30 min before dosing animals and unused portions were discarded. Gefitinib (a generous gift from AstraZeneca) was solubilized in 0.5% Tween-80 in sterile milli-Q water (vehicle) and kept at 4°C. Gefitinib formulation was prepared weekly. Combination treated mice were dosed first with gefitinib followed by RAD001 four hours later. Tumors were harvested 30 min after the last dose and cut into two parts: one part was frozen in liquid nitrogen (N_2_) for Western blot analysis and the second part was frozen in embedding medium and stored in -80°C for immunohistochemical processing. Animal protocols were approved by the University of British Columbia Animal Care Committee, and these studies were done in accordance with guidelines established by the Canadian Council on Animal Care.

### Alamar Blue and IN Cell 1000 screening assays

Cells were plated under standard serum conditions (10% FBS) in their respective media in triplicate wells/condition in 96-well flat bottom plates (Optilux, Falcon, Becton-Dickinson). MCF7-HER2 and JIMT-1 cells were plated at densities of 5,000 or 1,500 cells/well for 72 and 144 h drug incubations, respectively. SKBR3 cells were plated at 15,000 and 4000 cells/well for 72 and 144 h, respectively. Cells were allowed to adhere overnight. Next day the cells were treated with gefitinib, RAD001 and combination of both drugs at a fixed molar ratio over a broad dose range to establish growth curves for a 72 h and 144 h read-out. Stock solutions of 20 mM gefitinib and 20 mM RAD001 were prepared in DMSO and stored in -80°C. Gefitinib and RAD001 stocks were diluted in medium with decreasing percentage of DMSO and 10× concentrated drugs were added to cells. The final concentration of DMSO in vehicle and drug treated cells was standardized to 0.5% (vol/vol) and the final media volume in 96-well plate wells was 200 μl. After 72 or 144 h incubation, Alamar Blue (Invitrogen, Burlington, ON, CA) was added to one set of plates to evaluate cell viability. Fluorescence was measured using the FLUOstar OPTIMA plate reader (BMG Labtechnologies, Germany) with 544 nm excitation and 590 nm emission filters. A second set of plates was stained with DRAQ5 (Biostatus, Shepshed, UK) and ethidium homodimer (ETH; Molecular Probes, Invitrogen) followed by imaging with IN Cell 1000 Analyzer (GE Healthcare). Ten images per well were acquired with 10× objective. Data analysis strategies were supported by enterprise level servers. Images were analyzed with IN Cell 1000 Investigator software using the Multi Target Analysis (MTA) module and data were reported as the percentage of dead cells normalized to vehicle control by subtracting the percentage of dead cells in the DMSO control from the percentage of dead cells in treated cultures.

### Synergy Determination

Following drug treatment *in vitro*, the number of viable cells was measured using the Alamar Blue assay as described above. Alamar Blue measures mitochondrial activity which is lost upon cell death. The data obtained with the Alamar Blue assay were normalized to the vehicle control and expressed as % viability. Next, these data were converted to Fraction affected (Fa; range 0-1), where Fa = 0 represents 100% viability and Fa = 1 represents 0% viability) and analyzed with the CompuSyn™ program (Biosoft, Ferguson, MO) based upon the Chou and Talalay median effect principle [39]. This program calculates a combination index (CI) that is used to identify synergistic, additive, and antagonistic drug interactions.

### Flow cytometry

Cells were plated in their respective media containing 10% FBS in T25 flasks or 6 cm diameter culture dishes and allowed to adhere overnight. The next day cells were treated with the indicated agents. After 72 h, supernatant from treated cells was transferred to a 14 ml tube and combined with adherent cells harvested with 0.25% Trypsin EDTA. For cell cycle analysis cells were washed twice with PBS and 2 × 10^6 ^cells/sample were fixed in 1.8 ml cold (-20°C) 70% ethanol followed by 1 h incubation on ice and 24 h incubation in -20°C. Cells were then pelleted and stained in PBS buffer containing 50 μg/ml propidium iodide (PI, Molecular Probes, Invitrogen) with 1 mg/ml RNase A (Sigma-Aldrich) and 0.1% Triton X-100 (Bio-Rad, Richmond, CA) for 15 min at 37°C followed by 1 h incubation on ice. For apoptosis analysis cells were washed twice with Hank's media without phenol red and pellets were resuspended in Annexin-V buffer containing anti-Annexin-V-FITC antibody (Caltag Laboratories, Burlingame, CA). Samples were then incubated on ice for 30 min and counterstained with PI at a final concentration of 1 μg/ml. Flow cytometric analysis was performed with FACSCalibur flow cytometer and acquired data were analyzed with the Cellquest software (Becton-Dickinson, San Jose, CA).

### Western blotting

Cells were plated in T25 flasks or 6 cm culture dishes and after overnight adhesion treated with the indicated drugs. After 72 h cells were harvested in ice-cold PBS. Cell pellets were lysed in lysis buffer containing 50 mM Tris pH 7.4, 150 mM NaCl, 1% NP-40, 0.25% Na-deoxycholate, 1 mM EDTA, 0.1% SDS, and Mini Protease Inhibitor Cocktail tables (Roche Diagnostics, Mannheim, Germany). Tumors were homogenized in lysis buffer followed by sonication. After centrifugation (30 min at 13000 rpm) the protein concentration in the supernatant was quantified using the Pierce Micro BCA™ Assay Kit. 30 - 50 μg of total protein per sample was separated on precast 4-12% Bis-Tris gels (NuPage, Invitrogen) and transferred to NuPage 0.45 μm nitrocellulose membranes (Invitrogen). Membranes were blocked with 5% skim milk powder in TBS-T (150 mM NaCl, 50 mM Tris, 0.1% Tween-20, pH 7.4) and incubated overnight with primary antibodies in 5% BSA in TBS-T. The next day membranes were washed 3 times with TBS-T and incubated for 1 h with peroxidase-conjugated secondary antibodies (Promega) in TBS-T containing 5% skim milk. Membranes were washed 3 times with TBS-T and signals were detected by enhanced chemiluminescence (SuperSignal^® ^West Pico Chemiluminescent Substrate, Thermo Scientific) on BioMax Light Film (Kodak). All antibodies used for Western blot analysis were from Cell Signaling Technology (Beverly, MA). The following phospho-specific antibodies were used: P-EGFR (Tyr1086 antibody #2220), P-HER2 (Tyr1221/1222 antibody #2243 or Tyr1248 antibody #2247), P-ERK1/2 (Thr202/Tyr204 antibody #9101), P-AKT (Ser473 antibody #4060), P-70S6K (Thr389 antibody #9206), P-ribosomal protein S6 (Ser235/236 antibody #2211). The β-actin antibody (Sigma-Aldrich) was used as a loading control. Films with visualized protein bands were digitized and the optical density (OD) of bands was measured using UN-SCAN-IT graph and gel digitizing software (Silk Scientific, Inc.). After background subtraction the optical density (OD) value for each individual protein band was corrected for β-actin loading and normalized to the vehicle control expressed as l. Western blot analysis was repeated 2 - 3 times to assure consistency of the results.

### Immunohistochemistry, image acquisition and image analysis

10 μm cryosections were cut using a Cryostar HM560 (Microm International GmbH), air dried and then fixed in 50% (v/v) acetone/methanol for 10 min at room temperature. Endothelial cells were stained using a monoclonal antibody to PECAM/CD31 (BD Pharmingen) and fluorescent Alexa 647 secondary antibody (Invitrogen). Terminal deoxyribonucleotide transferase-mediated nick-end labeling (TUNEL) staining was used to label apoptotic cells (In situ Cell Death Detection kit, TMR red; Roche). Proliferating cells were stained using a polyclonal antibody to Ki67 (AbCam) followed by a peroxidase conjugated secondary antibody (Sigma-Aldrich) and metal-enhanced 3,3'-diaminobenzidine substrate (Pierce). Cell nuclei were labeled with Hoechst 33342 (8 ng/mL; Molecular Probes, Invitrogen) for 30 min at 37°C. At each stage of staining, whole tumor sections were imaged using a robotic fluorescence microscope, as previously described [40,41]. Automated tiling of adjacent microscope fields of view was completed to generate images of an entire tumor section at a resolution of 0.75 μm per pixel. All parameters stained on the same section were imaged separately using a monochrome camera and composite color images were generated using Adobe Photoshop (CS). Using NIH-Image http://rsb.info.nih.gov/nih-image and user-supplied algorithms, digital images were superimposed, aligned and cropped to tumor tissue boundaries with staining artifacts removed. Confluent necrosis was subsequently cropped from images and the degree of necrotic tissue calculated as the proportion of necrotic pixels relative to all pixels. ImageJ software applications http://rsb.info.nih.gov/ij/ and user-supplied algorithms were used to quantify the degree of staining above the thresholds determined to be > 10 standard deviations from background for CD31, TUNEL and Ki67, and data are reported as percent positive pixels of non-necrotic, viable tumor tissue. As a measure of tumor vascularization, the median distance of viable tissue to the nearest CD31-positive object (blood vessel) is reported (μm), such that a larger distance reflects a lower vascular density. Note that for CD31 analysis one JIMT-1 tumor was removed from each of the gefitinib and RAD001-gefitinib combination groups due to the presence of disproportionate necrosis; where only a narrow, avascular rim could be detected as viable tissue. To observe the location of proliferating cells in relation to blood vessels, Ki67 positive pixels were sorted based on their distance from CD31-positive vessels in 1.5 μm increments, and data are expressed as % positive Ki67 pixels relative to distance from vasculature (μm).

### Statistical analysis

One-way ANOVA was used to assess differences among the treatment groups with an unpaired t-test (GraphPad Prism version 5.00). The obtained p values were adjusted for multiple comparisons using the Benjamini-Hochberg procedure (R version 2.11.1). Differences were considered significant at *p *≤ 0.05.

## Results

### Gefitinib and RAD001 in combination synergistically decrease tumor cell viability *in vitro*

**Figure 1:**
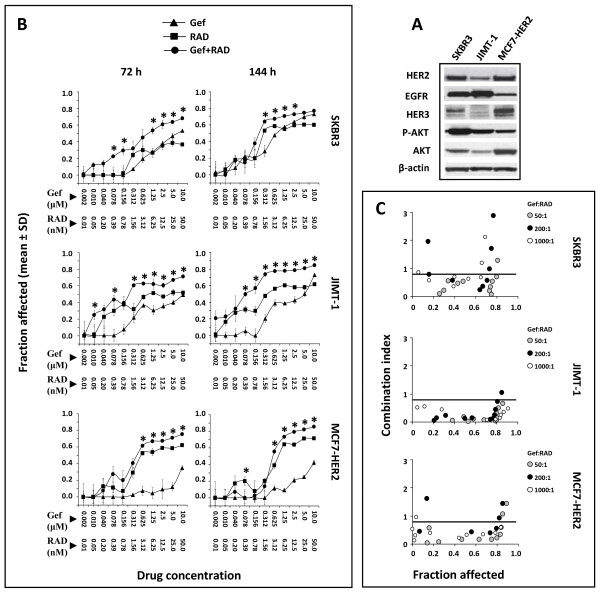
**Phenotypic analysis and *in vitro *sensitivity of SKBR3, JIMT-1 and MCF7-HER2 cells to gefitinib, RAD001, or the gefitinib and RAD001 combination**. **(A) **Expression of HER2, EGFR, HER3, P-AKT and AKT analyzed by Western blotting. **(B) **Cells were treated for 72 or 144 h with gefitinib (Gef), RAD001 (RAD) and the gefitinib and RAD001 combination at 200:1 molar ratio (Gef:RAD). Cell viability curves were plotted based on data obtained with an Alamar Blue assay and reported as Fa (fraction of cells affected by the treatment, where 1 is equivalent to 100% cytotoxicity). Each data point represents the mean ± SD of 3 wells. Asterisks above data points indicate significantly (p < 0.05) greater Fa in the combination treated cells compared to the single drugs. Achievable levels of gefitinib and rapamycin analogs reported in human blood are ~ 1 μM and 5-15 nM, respectively. All cell lines were screened 3 times with single drugs and 2-3 times with the drug combinations to assure consistency. The results presented are from a representative experiment. **(C) **The combination index (CI) was calculated with the CompuSyn™ software based on data derived from Alamar Blue assay completed in cells treated with the single drugs and drug combinations at the indicated molar ratios and plotted versus Fa. Each data point moving from left to right for any given ratio of gefitinib to RAD001 represents an effect of increasing drug concentrations. The data points below CI values of 0.8, denoted by a horizontal line on each plot, are indicative of synergistic interactions.

The efficacy of gefitinib, RAD001, or the combination was investigated in HER2 overexpressing TZ sensitive SKBR3 cells (ER negative, PTEN-positive, wild type PIK3CA gene encoding the p110-alpha catalytic subunit of PI3K), TZ sensitive MCF7-HER2 cells (ER positive, PTEN positive, mutated PIK3CA gene) and TZ resistant JIMT-1 cells (ER negative, PTEN positive, mutated PIK3CA gene) [12,38,42,43]. Overexpression of HER2 in SKBR3 and JIMT-1 cells is due to endogenous gene amplification while the MCF7-HER2 cells have been transfected with the erbB2 gene driven by the CMV promoter. The three cell lines co-express EGFR, with the highest protein levels noted in JIMT-1 cells (Figure 1A). The expression of HER-3 protein was highest in MCF7-HER2 and relatively low in SKBR3 and JIMT-1 cells and P-AKT was constitutively expressed in all three cell lines (Figure 1A). Using an Alamar Blue assay to assess cell viability, we determined the cell fraction affected (Fa) by each treatment following a 72 h or 144 h exposure to the drugs alone and in combination. After 72 h viability decreased in response to a broad range of gefitinib or RAD001 concentrations in all treated cell lines (Figure 1B). Fa was improved after increasing drug exposure to 144 h, with the greatest improvement in SKBR3 cells reflecting their relatively slow proliferation rates (Figure 1B). The inhibitory concentration of gefitinib and RAD001 which elicited a 50% decrease in cell viability (IC_50_), was calculated using CompuSyn™ software based on the Alamar Blue data. In JIMT-1 and MCF7-HER2 cells the IC_50 _values for RAD001 after 72 h reached 19 ± 2.2 and 10.7 ± 8.1 nM (mean ± SE) respectively; an IC_50 _for RAD001 was not achieved in SKBR3 at this time point. After 144 h treatment the IC_50 _values for RAD001 were 5.9 ± 3.7, 6.8 ± 2.5, 5.4 ± 1.9 nM (mean ± SE) in SKBR3, JIMT1 and MCF7-HER2 cells respectively, thus demonstrating that all three cell lines have a similar sensitivity to RAD001. The dose response curves for RAD001 showed a concentration dependent reduction in cell viability up to ~ 3 - 6 nM. Higher doses of RAD001 resulted in only a small incremental rise in the Fa, suggesting saturation of RAD001 effects (Figure 1B). The IC_50 _values for gefitinib after 72 h treatment were 4.3 ± 1.2 μM (mean ± SE) in SKBR3 cells and > 10 μM in JIMT-1 and MCF7-HER2 cells; after 144 h the IC_50 _values were 1.6 ± 1, 5 ± 0.5 and > 10 μM (mean ± SE) respectively in SKBR3, JIMT-1 and MCF7-HER2 cells. These results showed that SKBR3 cells were sensitive, JIMT-1 cells were moderately resistant and MCF7-HER2 cells were exceptionally resistant to gefitinib.

Guided by the IC_50 _values determined for the single drugs, the gefitinib and RAD001 combinations were evaluated at the 50:1, 200:1 and 1000:1 (Gef:RAD) fixed molar ratios. The effects of the gefitinib and RAD001 combination on cell growth assessed at 200:1 molar ratio (Gef:RAD) are presented in Figure 1B as an example of the data showing that the combination exerted a significantly greater (p < 0.05) growth inhibition in all three cell lines when compared to the single drugs at the dose points indicated by asterisks. These points include therapeutically relevant gefitinib and RAD001 concentrations that can be achieved in human blood and which were previously reported as ~1 μM for gefitinib [44] and ~ 5-15 nM for rapamycin and derivatives [45]. The dose response data were then evaluated with the CompuSyn™ program which applies the median effect methodology developed by Chou and Talalay [39] to assess drug-drug interactions. This algorithm estimates a combination index (CI) for each data point based on the results expected from each of the single agents. If the experimental effects of the combination are greater than expected, then the CI value will be less than 1. If the experimental effects are less than expected the CI value will be greater than 1. CI values of 0.8 or less are indicative of robust synergistic interactions. Figure 1C shows that after 144 h treatment with the gefitinib and RAD001 combination, a CI < 0.8 was reported over a wide range of Fa regardless of the drug:drug ratio used, reflecting synergistic interactions between gefitinib and RAD001. Importantly, these data underline the fact that the gefitinib and RAD001 combination is equally effective in HER2 overexpressing cells irrespective of their intrinsic resistance to gefitinib or TZ.

### Cytotoxic and cytostatic effects of gefitinib, RAD001 and the combination *in vitro*

**Figure 2:**
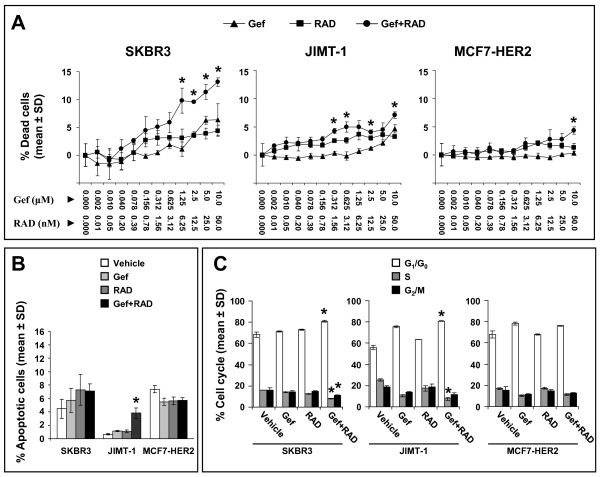
**Assessment of cell death, apoptosis and cell cycle in SKBR3, JIMT-1 and MCF7-HER2 cells treated with gefitinib, RAD001 and the gefitinib and RAD001 combination used at 200:1 (Gef:RAD) molar ratio**. **(A) **Assessment of cell death by HCS. Cells were seeded in 96-well plates and treated with indicated drug concentration. After 72 h cells were stained *in situ *with DRAQ5 (stain for viable cells) and ethidium homodimer (stain for dead cells) and images were acquired with IN Cell 1000. The imaging data were analyzed with the IN Cell 1000 Investigator software and the results are expressed as percentage of dead cells relative to DMSO control. Asterisks above data points indicate a significantly (p < 0.05) greater percentage of dead cells in the combination treated cells compared to cells treated with gefitinib or RAD001 alone. Achievable levels of gefitinib and rapamycin analogs reported in human blood are ~ 1 μM and 5-15 nM, respectively. Each data point represents the mean ± SD from 3 replicate wells. Data from representative experiments are shown. **(B - C) **Flow cytometric analysis of apoptosis (B) and cell cycle (C) in cells treated with 1 μM gefitinib (Gef), 5 nM RAD001 (RAD) or the combination of both drugs at 200:1 (Gef:RAD) molar ratio. Each bar represents a mean ± SD from 3 replicate samples. Asterisks indicate a significant difference (p < 0.05) between cells treated with the gefitinib and RAD001 combination compared to the single drugs. Representative experiments are shown.

The mechanisms associated with the synergistic growth inhibition *in vitro *by the gefitinib and RAD001 combination were further investigated using high content screening (HCS) methods to quantitate viable and dead cells in cultures stained *in situ *with DRAQ5 and ETH. The HCS data showed that 72 h treatment with gefitinib plus RAD001 caused a significant (p < 0.05) increase in cytotoxicity in SKBR3 and JIMT-1 cells at multiple doses (Figure 2A). In MCF7-HER2 cells growth inhibition by the combination (as shown in Figure 1B) was accompanied by cytotoxic effects only when drugs were used at high and physiologically irrelevant concentrations (10 μM gefitinib and 50 nM RAD001) [44,45]. After 144 h treatment with the gefitinib and RAD001 combination, an additional increase in cell death took place in SKBR3 and JIMT-1 cells but not in MCF7-HER2 cells (data not shown).

To further investigate the mechanisms of action of the gefitinib and RAD001 combination we performed flow cytometric analysis of apoptosis and cell cycle. Cells were treated for 72 h with 1 μM gefitinib, 5 nM RAD001 or the combination of both drugs at 200:1 molar ratio (Gef:RAD). There was no significant increase (p > 0.05) found in Annexin positive PI negative apoptotic cells in SKBR3 or MCF7-HER2 cultures treated with the combination compared to treatment with the single drugs at corresponding concentrations (Figure 2B). In JIMT-1 cells treated with the combination there was a 3-fold increase in apoptosis relative to gefitinib or RAD001 treatment alone (p < 0.05); however, the absolute percentage of apoptotic cells in the combination treated cultures was only 4% compared to 1% in the single drug treated cultures.

Cells treated in an identical manner as described above were then analyzed for changes in cell cycle. The data summarized in Figure 2C show that the gefitinib and RAD001 combination significantly increased (p < 0.05) the proportion of cells in G_1_/G_0 _and reduced the S phase fraction in SKBR3 and JIMT-1 cells, when compared to either of the drugs alone. No significant changes in cell cycle (p > 0.05) were observed in MCF7-HER2 cells (Figure 2C).

### Gefitinib and RAD001 in combination decrease activity of the mTOR pathway *in vitro*

**Figure 3:**
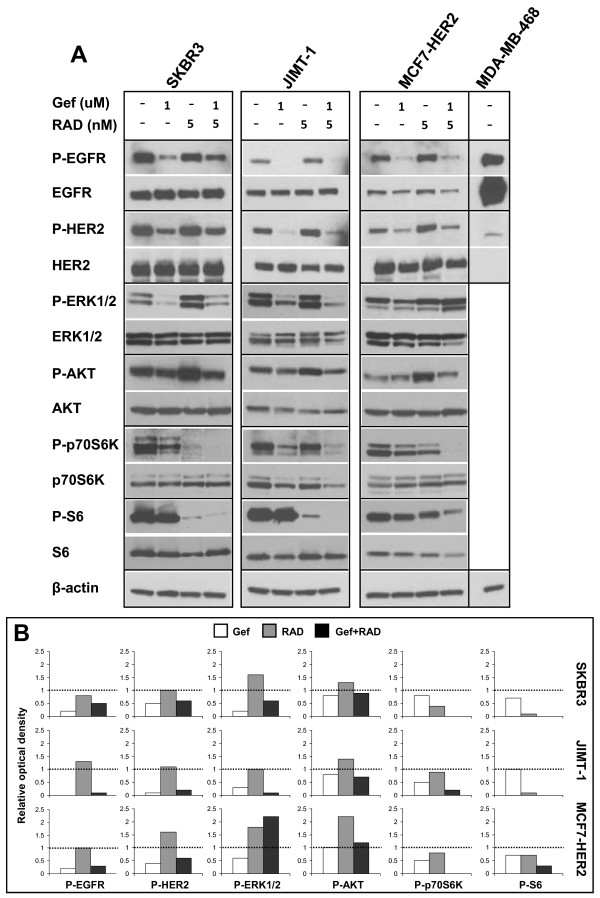
***In vitro *molecular responses to gefitinib, RAD001 and the gefitinib and RAD001 combination**. SKBR3, JIMT-1 and MCF7-HER2 cells were treated for 72 h with indicated concentrations of gefitinib (Gef), RAD001 (RAD) or the combination of both drugs at a 200:1 (Gef:RAD) molar ratio. Cell lysates were prepared from harvested cells and expression of phospho- and corresponding total protein were analyzed with Western blotting. **(A) **Representative images of protein bands. **(B) **The expression of phosphorylated proteins, as shown in (A) was measured by densitometry and normalized to the vehicle control expressed as 1 and denoted by a dotted line in each histogram. β-actin was used to control for equal loading. The MDA-MB-468 cell line with a high EGFR and negligible HER2 expression was used to control for specificity of the anti P-EGFR and P-HER2 antibodies.

To investigate the molecular changes in HER2 overexpressing breast cancer cells after treatment with the gefitinib and RAD001 combination, we analyzed the expression and phosphorylation of proteins relevant to EGFR, HER2 and mTOR signaling. The focus of this investigation was to characterize more stable long-term changes that occurred after 72 h of treatment rather than assessing immediate effects that may be transient in nature. The results summarized in Figure 3 show that treatment with 1 μM gefitinib lowered the levels of P-EGFR, P-HER2, P-ERK1/2 and P-p70S6K compared to vehicle treated cells in all three cell lines; inhibition of P-AKT was observed in SKBR3 and JIMT-1 and inhibition of P-S6 in SKBR3 and MCF7-HER2 cells (Figure 3A and 3B). RAD001 inhibited activity of mTOR downstream targets (P-p70S6K and P-S6) but increased P-AKT levels relative to the controls in all three cell lines (Figure 3A and 3B). RAD001 also caused upregulation of P-EGFR in JIMT-1 cells, P-HER2 in MCF7-HER2 cells and P-ERK1/2 in SKBR3 and MCF7-HER2 cells. When gefitinib and RAD001 were used in combination there was a considerably greater reduction noted in P-p70S6K and P-S6 compared to the single drugs which was consistent in all three cell lines. A further decrease in P-ERK1/2 and P-AKT by the combination took place only in JIMT-1 cells (Figure 3A and 3B). Gefitinib, when added to RAD001, was also able to counteract RAD001 induced hyperphosphorylation of EGFR, HER2, ERK1/2 and AKT in various cell lines, except for ERK1/2 in MCF7-HER2 cells (Figure 3A and 3B). These data suggest that inhibition of the mTOR pathway by RAD001 is enhanced in the presence of gefitinib, which also for the most part prevented RAD001 induced increases in certain phosphoproteins. Interestingly, in MCF7-HER2 cells treated with gefitinib and the combination, a reduction in P-EGFR, P-HER2 and P-S6 was accompanied by lower levels of total EGFR, HER2 and S6 (Figure 3A), and in combination treated JIMT-1 cells a decrease in P-p70S6K also occurred in parallel to decreased p70S6K (Figure 3A).

### Gefitinib and RAD001 in combination impede growth of established TZ sensitive and TZ resistant tumors

**Figure 4:**
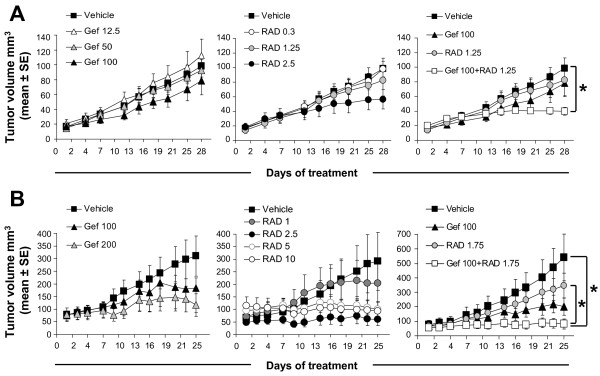
**Efficacy of gefitinib, RAD001 and the combination in JIMT-1 and MCF7-HER2 tumors**. S.c. tumors were established in Rag2M mice and animals were treated for 28 days (JIMT-1) or 25 days (MCF7-HER2) with the indicated drugs. Growth curves of **(A) **JIMT-1 and **(B) **MCF7-HER2 tumors (n = 6 mice/treatment group) plotted based on tumor volume. Numbers in figure legends indicate drug doses in mg/kg of body mass. Asterisks represent a significant (p < 0.05) difference between the average tumor volume on the last day of treatment between indicated groups.

The *in vitro *data presented thus far strongly suggest that the gefitinib and RAD001 combination exerts beneficial therapeutic effects in HER2 overexpressing breast cancer cell lines, irrespective of their TZ or gefitinib sensitivity status. To test the efficacy of this combination *in vivo*, animals bearing established JIMT-1 and MCF7-HER2 tumors were treated for 28 and 25 days, respectively, with gefitinib, RAD001 or a combination of the two drugs. The results summarized in Figure 4A (left panel) show that JIMT-1 tumors exhibited no change in growth rate when tumor bearing mice were treated with 12.5 or 50 mg/kg gefitinib, but at 100 mg/kg a moderate 1.3-fold reduction in tumor volume relative to controls was observed (p > 0.05). When mice bearing established JIMT-1 tumors were treated with 1.25 mg/kg and 2.5 mg/kg RAD001, there was a 1.2-fold and 1.7-fold decrease in tumor volume, respectively, when measured on the last day of treatment, but again, tumor volume was not significantly different (p > 0.05) from tumors in the vehicle treated group (Figure 4A, middle panel). For the combination treatment, doses of 100 mg/kg gefitinib and 1.25 mg/kg RAD001 were selected, as they provided a sub-optimal therapy. This approach has a strategic advantage over using the maximum tolerated doses of single drugs in combination experiments since it helps to clearly demonstrate whether combining drugs provides an improvement in reducing tumor volume. Treatment of animals bearing JIMT-1 tumors with the gefitinib and RAD001 combination caused a significant (p < 0.05) 2.5-fold decrease in tumor volume on the last day of treatment relative to controls; however, these tumors were not significantly different compared to tumors harvested from animals treated with gefitinib or RAD001 alone (p > 0.05) (Figure 4A, right panel). MCF7-HER2 tumors were more sensitive to gefitinib and RAD001 than JIMT-1 (Figure 4B). Increasing the gefitinib dose to 200 mg/kg and RAD001 above 2.5 mg/kg resulted in a greater therapeutic effect represented by stable disease rather than tumor regression in animals bearing MCF7-HER2 tumors (Figure 4B, left and middle panels). Gefitinib used at 100 mg/kg and RAD001 used at 1.75 mg/kg reduced tumor volume by 2.7-fold and 1.6-fold, respectively, relative to the vehicle control group but these differences were not statistically significant (p > 0.05) (Figure 4B, right panel). However, the average MCF7-HER2 tumor volume on the last day of treatment in the combination treated group was significantly smaller (p < 0.05) than in the control or RAD001 group (6.7-fold and 4.3-fold reduction, respectively). In contrast, the difference between the combination and gefitinib treated tumors was not statistically significant (p > 0.05) (Figure 4B, right panel). These data show that the combination treatment was more potent than the single drugs when compared to vehicle treated controls. Importantly, the combination prevented further growth of TZ sensitive and resistant tumors. The synergy analysis based on the median effect methodology developed by Chou and Talalay [39] could not be performed on the *in vivo *data because the combination was only tested at one dose of gefitinib.

It should be noted that none of the treatment regimens caused any considerable body weight loss in animals (p > 0.05). Detailed animal health monitoring data (not reported here) suggested that gefitinib and RAD001 were well tolerated at the doses used, whether the drugs were used alone or in combination. It is important to note that we also tested sensitivity of JIMT-1 tumors to TZ in Rag2M mice. The results of this study presented in "Additional file 1" show that treatment with TZ (up to 20 mg/kg given twice weekly (i.p.) over the course of 27 days) did not cause inhibition of tumor volume (p > 0.05), thus, confirming the resistance of JIMT-1 cells to TZ, as previously determined by others [38].

### Effects of gefitinib, RAD001 and the combination on tumor tissue characteristics

**Figure 5:**
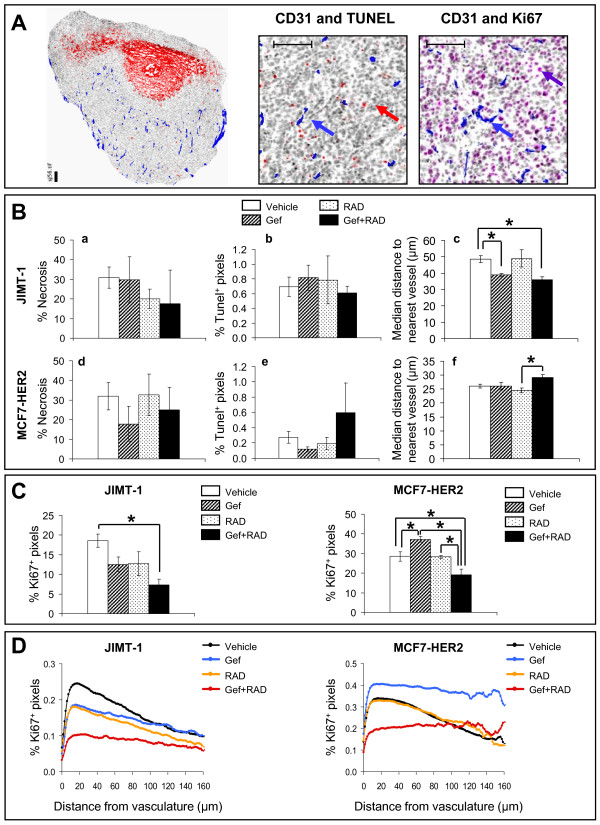
**Immunohistochemical analysis of tumor tissue sections**. **(A) **Left panel: representative image of a whole tumor section stained with anti-CD31 antibody (blue), Hoechst 33342 (gray), and TUNEL (red) to label vasculature, nuclei and apoptosis, respectively. Vast red areas in tumor tissue represent continuous apoptosis regions, referred to as "necrosis". Middle panel: a magnified image of a viable area in tumor tissue section shown in the left panel; a blue arrow indicates a CD31 positive blood vessel; a red arrow indicates a TUNEL-positive apoptotic cell. Right panel: representative image of a viable area in the tumor tissue section stained with Hoechst 33342 (grey), anti-CD31 antibody to label blood vessels (a blue arrow) and anti-Ki67 antibody to label proliferating cells (a purple arrow). Scale bars: 150 μM. **(B) **Quantification of necrosis (a and d), apoptosis (b and e) and vascularization (c and f) in tumor tissue sections. Apoptosis is shown as % TUNEL-positive pixels within viable tumor tissue. The median distance from viable tumor tissue to the nearest vessel reflects microvessel density; the greater the vessel distance, the lower the microvessel density and *vice versa*. **(C) **The percentage of Ki67-positive pixels in viable tumor tissue. Bars in B and C represent the mean ± SE calculated from 4-5 tumors per treatment group and asterisks denote a statistically significant difference (p < 0.05) between the indicated groups. **(D) **Mean distribution of Ki67 positive pixels relative to CD31-stained vessels mapped in tumor tissue sections. The IHC analysis was completed twice using different cryosections to assure consistency of the results.

Immunohistochemistry (IHC) based tumor tissue mapping techniques were used to investigate changes in JIMT-1 tumors harvested from animals treated for 28 days with 100 mg/kg gefitinib, 1.25 mg/kg RAD001 or the gefitinib and RAD001 combination and in MCF7-HER2 tumors harvested from animals treated for 25 days with 100 mg/kg gefitinib, 1.75 mg/kg RAD001 or the combination. The area of confluent TUNEL-positive tissue, herein described as "necrosis" (Figure 5A, left panel) and TUNEL staining within regions of viable tumor tissue, indicative of apoptotic cells, along with CD31 staining and proliferation status (Ki67) of tumor tissue were assessed (Figure 5A, middle and right panel). The results indicate that the mean level of necrosis (Figure 5B, a and 5d) and apoptosis (Figure 5B, b and 5e) did not differ between treatment groups (p > 0.05) in JIMT-1 and MCF7-HER2 tumors. Because gefitinib and RAD001 have been reported to exert anti-angiogenic effects [21,36,46,47], we also investigated possible changes in tumor vascularization. An overall higher vessel density was seen in the MCF7-HER2 tumors where the median distance of tumor tissue to the nearest CD31-positive object (vessel) was ~ half that of the JIMT-1 tumors (Figure 5B, c and 5f). The median distance of tumor tissue to the nearest CD31-positive vessel in JIMT-1 tumors derived from animals treated with gefitinib was significantly (p < 0.05) decreased compared to vehicle control suggesting an increase in vascularization. No changes were noticed in tumors derived from animals treated with RAD001 alone (p > 0.05) and the combination for the most part reflected the effects of gefitinib (Figure 5B, c). In MCF7-HER2 tumors, gefitinib, RAD001 and the combination did not produce any significant (p > 0.05) changes in vascularization relative to the vehicle control (Figure 5B, f).

Evidence presented in Figure 5B suggests that tumor growth inhibition by the gefitinib and RAD001 combination is not associated with an increase in either necrosis or apoptosis. To find out if the growth inhibition is a result of altered proliferation rates of tumor cells, we analyzed the expression of Ki67 in tumor sections. Figure 5C (left graph) shows that in JIMT-1 tumors the combination caused a significant reduction in Ki67 expressing tissue (p < 0.05; 2.5-fold) compared to vehicle treated controls. This is in contrast to the single drugs which did not produced a significant reduction in Ki67 relative to the control group (p > 0.05). In MCF7-HER2 tumors the expression of Ki67 was significantly lower (p < 0.05) in the combination treated group compared to vehicle (1.5-fold decrease) and also to the single drug treated groups (1.9-fold and 1.5-fold decrease compared to gefitinib and RAD001 group, respectively) (Figure 5C, right graph).

When these differences were further investigated by mapping the micro-regional distribution of Ki67 positive pixels relative to CD31-stained vessels, more specific detail was obtained. In JIMT-1 tumors, decreased Ki67 staining was observed in all treatment groups relative to controls and at all distances from vasculature, with the combination showing the greatest effect at tumor tissue in close proximity to vessels (Figure 5D, left graph). A different pattern was observed in MCF7-HER2 tumors, where RAD001 showed no major change relative to controls but gefitinib alone caused a remarkable increase in proliferation both at near proximity and > 100 μM from the vessels. In MCF7-HER2 tumors the combination treatment was the only regimen to affect a drastic decrease in proliferation in tissue proximal to vasculature which, similarly to gefitinib treatment alone, did not decrease further with growing distance from vessels (Figure 5D, right graph). In summary, these data provide evidence that gefitinib and RAD001 when used in combination *in vivo *do not increase cytotoxicity but interact to increase cytostasis, with greater effects in tumor tissue proximal to vasculature.

### Gefitinib and RAD001 in combination decrease levels of P-EGFR and inhibit the mTOR pathway *in vivo*

**Figure 6:**
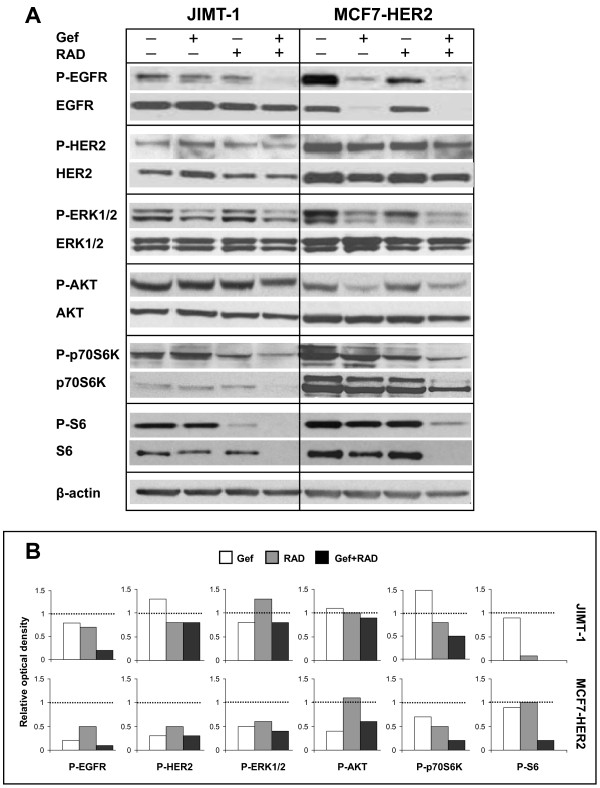
***In vivo *molecular responses to gefitinib, RAD001 and the gefitinib and RAD001 combination**. Western blot analysis of phospho- and corresponding total protein levels in tissue lysates derived from JIMT-1 and MCF7-HER2 tumors harvested from Rag2M mice at the end of treatment. Animals bearing JIMT-1 tumors were treated with 100 mg/kg gefitinib (Gef), 1.25 mg/kg RAD001 (RAD) and the combination of both drugs and animals bearing MCF7-HER2 were treated with 100 mg/kg gefitinib, 1.75 mg/kg RAD001 and the combination of both drugs. Protein concentration in tumor tissue lysates from individual tumors was adjusted to 50 mg/ml and an equal volume of each individual tumor lysate was pooled for loading gels. Results from six pooled tumor lysates per lane are shown. **(A) **Representative images of protein bands. **(B) **The phosphoprotein levels shown in (A) relative to β-actin were determined by densitometry and normalized to the vehicle control expressed as 1 and denoted by a dotted line in each histogram.

To assess molecular changes in JIMT-1 and MCF7-HER2 tumors harvested from treated animals, tumor tissue lysates were analyzed with Western blotting. The results summarized in Figure 6 show protein bands (Figure 6A) and optical density of each band corrected for β-actin expression and normalized to the vehicle control (Figure 6B). Gefitinib treatment resulted in decreased P-EGFR, P-ERK1/2 and P-S6 levels, relative to vehicle controls, in MCF7-HER2 and JIMT-1 tumors (Figure 6A and 6B). In addition, gefitinib caused a decrease in P-HER2, P-AKT and P-p70S6K levels in MCF7-HER2 tumors (Figure 6A and 6B). Targeting the mTOR pathway with RAD001 brought about a decrease in P-p70S6K and, interestingly, also a decrease in P-EGFR and P-HER2 levels in JIMT-1 and MCF7-HER2 tumors. P-S6 was inhibited only in JIMT-1 tumors and there were no noteworthy changes in P-AKT after treatment with RAD001 (Figure 6A and 6B). Addition of gefitinib to RAD001 resulted in greater inhibition of P-EGFR, P-p70S6K and P-S6 in both JIMT-1 and MCF7-HER2 tumors (Figure 6A and 6B) compared to the single drugs. In contrast, the combination exerted a reduction in P-ERK1/2 and P-AKT that was very moderate and tumor type specific (Figure 6B). The results also show that total EGFR, HER2, p70S6K and S6 protein expression in tumors treated with the combination was decreased in parallel to levels of corresponding phosphoproteins in JIMT-1 and MCF7-HER2 tumors (Figure 6A). Gefitinib also caused a drastic decrease in total EGFR and somewhat smaller decrease in HER2 in MCF7-HER2 tumors (Figure 6A). Overall, these data suggest that the gefitinib and RAD001 combination *in vivo *reduces activity of EGFR, p70S6K and S6 through inhibiting function as well as decreasing expression of these proteins.

## Discussion

New treatment strategies are needed for patients with advanced HER2-positive breast cancers due to very limited therapeutic options available for fighting this disease. Encouraged by previously published reports demonstrating synergistic interactions between EGFR and mTOR inhibitors in various cancers [34-36] we investigated the activity of the EGFR targeted drug gefitinib used in combination with a rapamycin analog, RAD001, in HER2 overexpressing and EGFR co-expressing breast cancer models with TZ sensitive or resistant phenotypes [12,38,42,43]. The rationale to study this combination in HER2 positive breast cancers has also been strengthened by a recent investigation of Miller *et al. *who demonstrated that inhibition of PI3K and mTOR are required for the growth inhibitory effects of HER2 antagonists in HER2 overexpressing breast cancer and that inhibition of mTOR is an effective therapeutic strategy in TZ resistant breast cancer models [48].

Our data showed that while SKBR3 cells were sensitive to gefitinib, JIMT-1 and MCF7-HER2 cells were gefitinib resistant; however, RAD001 was capable of sensitizing these cells to gefitinib. It is interesting to note that both JIMT-1 and MCF7-HER2 cell lines harbor PIK3CA mutations which have been associated with acquired resistance to EGFR kinase inhibitors but can also predict sensitivity towards mTOR inhibition [43,49,50]. Together with our data, this may suggest that RAD001 is able to reverse gefitinib resistance in PIK3CA mutant tumors. Our data indicate that *in vitro *gefitinib and RAD001 interact in a synergistic fashion, as shown by a mathematical model developed by Chou and Talaly [39] and this synergy did not appear to be drug ratio dependent. The *in vivo *efficacy of gefitinib and RAD001 was also greatly improved when these drugs were used in combination. Further validation of our results in other models of HER2 overexpressing and TZ resistant breast cancers such as MDA-MB-453, MDA-MB-361 or UACC893 would be crucial in order to determine if this combination is broadly effective in TZ resistant cancers. However, our results obtained using the JIMT-1 model do give an indication that the gefitinib and RAD001 combination was able to effectively target the cellular machinery that is indispensable for cancer cell growth despite the existence of multiple mechanisms contributing to the extreme TZ resistance of this cell line [38,42]. It should be noted that while the combination treatment did not result in regression of established tumors, this could be a consequence of our experimental design. We opted to use doses of gefitinib and RAD001 that on their own did not produce statistically significant (p > 0.05) reduction in tumor volume relative to vehicle treated controls, so that inhibition of tumor growth by the combination would be evident. Consequently, gefitinib given at 100 mg/kg resulted in a more potent reduction in MCF7-HER2 tumor volume than anticipated on its own, thus making the effect of the combination very modest.

The data obtained based on analysis of multiple endpoints after 72 h treatment suggest a contribution of cytostasis in the presence (in SKBR3 and JIMT-1 cells) or absence (in MCF7-HER2 cells) of cytotoxicity to the synergy between gefitinib and RAD001 *in vitro*. Treatment with the combination induced apoptosis only in JIMT-1 cells; however, it should be noted that Annexin V is a marker for the early apoptotic event so apoptosis may not be detected in SKBR3 and MCF7-HER2 cells after 72 h. Thus, a contribution of apoptosis to cytotoxicity at earlier time points is possible. Our findings are consistent with other reports demonstrating that gefitinib and RAD001 are cytostatic in nature [12,14,25,45] and that the degree of cytotoxicity triggered by these drugs is a cell type dependent phenomenon [14,48]. This perhaps reflects PIK3CA or other mutations in genes controlling cell growth, proliferation and survival [43]. While the enhancement of cytostasis seen after 72 h in the combination (1 μM gefitinib with 5 nM RAD001) treated SKBR3 and JIMT-1 cells was confirmed by increased G_1_/G_0 _cell cycle arrest and decreased S phase relative to the single drugs, the combination failed to induce significant cell cycle changes in MCF7-HER2 cells despite growth inhibition in the absence of cytotoxicity. It has been reported that the parental MCF7 cell line expresses high levels of activated p70S6K and cyclin D1 [51] which may have contributed to somewhat obscure cell cycle regulation, possibly resulting in longer time required to complete a cell cycle or perhaps a transient cell cycle block that was resolved before 72 h. Increased cytostasis by the gefitinib and RAD001 combination in the absence of increased cytotoxicity was also found *in vivo *in JIMT-1 and MCF7-HER2 tumor xenografts. This may explain why the combination stabilized tumor growth and did not cause tumor regression. Interestingly, gefitinib increased levels of Ki67-positive cells in MCF7-HER2 tumors. These proliferating cells were present at similar frequency in proximal and longer (> 100 μM) distances from the blood vessels suggesting that tissue perfusion in gefitinib treated tumors was perhaps improved. In support, our previous study found that MCF7-HER2 tumors treated with gefitinib contain a greater proportion of functional Hoechst 33342 perfused vessels and this correlated with significantly increased tumor tissue oxygenation resulting in fewer hypoxic cells present [52]. The study of Lu et al. also showed that positive therapeutic responses of cancer cells to EGFR-targeted therapy with cetuximab and gefitinib are associated with downregulation of hypoxia-inducable-factor-1α (HIF-1 α) [53]. Furthermore, Hardee et al. reported that blockade of HER2 signaling in MCF7-HER2 tumors with TZ improved tumor tissue oxygenation and vascular architecture along with increased microvessel density [54]. Thus, we speculate that gefitinib treatment perhaps resulted in vessel normalization. In turn, improved vessel functionality could be responsible for more efficient delivery of drugs to tumor tissue and increased cytostasis. This may explain why MCF7-HER2 tumors were more sensitive to gefitinib than JIMT-1 tumors, even though we found the opposite to be true *in vitro *in MCF7-HER2 and JIMT-1 cells.

The most striking and consistent therapeutic effect of the combination noted *in vitro *and *in vivo *was greater inhibition of the mTOR pathway reflected by decreased P-p70S6K and P-S6 levels relative to the effects of the single drugs. These changes strongly correlated with better efficacy of the combination treatment. Accordingly, several reports suggested that P-p70S6K can be considered as a biomarker for monitoring treatment outcomes in patients receiving mTOR inhibitors [25,45,55,56]. While the combination did not decrease P-EGFR levels *in vitro *compared to the single drugs, enhanced inhibition of P-EGFR by the combination *in vivo *appeared to be a consistent molecular event in JIMT-1 and MCF7-HER2 tumors. This can be attributed to inhibition of P-EGFR by both gefitinib and RAD001. The latter effect was not reported in other studies and the mechanisms involved are unclear at this point. Improved inhibition of P-EGFR by the combination *in vivo *may certainly play a role in downregulation of the mTOR pathway but how this is achieved without robust inhibition of AKT and ERK1/2 activity remains a question for further research. Interestingly, the *in vivo *reduction in P-EGFR, P-HER2, P-p70S6K and P-S6 levels was mirrored by decreases in total expression of the corresponding proteins in different treatment groups. Similar correlations were observed *in vitro *for selected proteins in gefitinib and/or combination treated MCF7-HER2 and JIMT-1 cells. These data suggest that inhibition of translation rates or perhaps changes in post-translational events regulating the expression of EGFR, HER2, p70S6K and S6 proteins may have contributed to decreased signaling in addition to direct effects on protein phosphorylation. In contrast, the expression of ERK1/2 and AKT *in vivo *was not altered after different treatments indicating that changes in phosphorylation levels actually reflected activation status of these proteins.

It should be noted that despite greatly improved inhibition of the mTOR pathway by the gefitinib and RAD001 combination our data suggest lack of or only moderate inhibitory effects of the combination on P-AKT levels. This result can be explained by a RAD001 mediated negative feedback loop. It has been demonstrated that inhibition of mTORC1 by rapamycin analogs initiates p70S6K-dependent feedback signaling resulting in stimulation of mTORC2 and phosphorylation of AKT on Ser473 [24-30]. Our *in vitro *data show that after 72 h RAD001 increased P-AKT levels in all three cell lines, but addition of gefitinib to RAD001 was able to counteract this effect. RAD001 also enhanced P-ERK1/2 levels in SKBR3 and MCF7-HER2 cells and in JIMT-1 tumors and these results are in agreement with studies showing activation of ERK1/2 through a PI3K-dependent feedback loop following inhibition of mTORC1 in some human cancers [57]. Again, addition of gefitinib to RAD001 counteracted activation of ERK1/2 in SKBR3 cells and in JIMT-1 tumors. Nonetheless, absence of robust inhibition of AKT and ERK1/2 activity *in vivo *after treatment with the combination is of concern since it may provide cancer cells with a survival advantage and lead to development of drug resistance and escape from cytostasis which consequently would limit treatment efficacy [3-5,18,58,59]. Likewise, other investigators have shown that targeting HER2 and mTOR using the TZ and RAD001 combination inhibits growth of HER2 overexpressing cancers to a greater extent than single agents, but this treatment did not further reduce P-AKT or P-ERK1/2 levels, when compared to the single drug effects [48]. Thus, combining drugs that inhibit function of EGFR/HER2 with dual PI3K/mTOR and MEK pathway inhibitors in order to abolish compensatory mechanisms may eliminate cancer cell survival and perhaps improve therapeutic effects in HER2-positive breast cancers [59-62].

## Conclusions

In summary, we showed that the gefitinib and RAD001 combination is therapeutically effective in HER2 overexpressing breast cancers irrespective of their TZ or gefitinib sensitivity status. The combination increased inhibition of cancer growth *in vitro *and *in vivo*. The beneficial therapeutic effects of gefitinib and RAD001 when used in combination appear to be related to ability of drugs to achieve efficient inhibition of mTOR (*in vitro *and *in vivo*) and EGFR (*in vivo*) signaling while at the same time eliminating negative feedback effects. Because the gefitinib and RAD001 combination shows a favorable safety profile *in vivo *and both drugs are approved for human use, this combination could be rapidly translated into opportunities in the clinic.

## Competing interests

The authors declare that they have no competing interests.

## Authors' contributions

WHD designed experiments, performed flow cytometric analysis, analyzed data, interpreted results and wrote the draft of the manuscript. SAW performed Western blot experiments and helped with the final draft of the manuscript. MAQ supported the computer platform to perform HCS data analysis. LYW performed cell culture, HCS experiments and assisted in animal experiments. YF and GK performed Western blot experiments. AIK analyzed the HCS data. JHEB performed and interpreted the IHC and tumor mapping and AIM contributed to tumor mapping data interpretation. DM performed animal experiments. KAG and MBB conceived the study, participated in its design and helped with the manuscript draft. All authors read and approved the final manuscript.

## Pre-publication history

The pre-publication history for this paper can be accessed here:

http://www.biomedcentral.com/1471-2407/11/420/prepub

## Supplementary Material

Additional file 1 **Efficacy of TZ in JIMT-1 tumors**. JIMT-1 xenografts (s.c.) were established in Rag2M mice and animals were treated with vehicle (saline) or 2.5, 5, 10 and 20 mg/kg trastuzumab (TZ) (n = 6 animals/treatment group). Treatment was initiated on day 21 and administered as intra-peritoneal injections (i.p.) twice weekly for 4 weeks. Click here for file
