# Supplementary figures and images for: The combination of gefitinib and RAD001 inhibits growth of HER2 overexpressing breast cancer cells and tumors irrespective of trastuzumab sensitivity
Source: BMC Cancer. 2011 Oct 1;11:420. doi: 10.1186/1471-2407-11-420 (PMC3207940; doi:10.1186/1471-2407-11-420)

**Tumor volume mm<sup>3</sup>  
(mean  $\pm$  SE)**

- Vehicle
- △ TZ 2.5
- △ TZ 5
- △ TZ 10
- ▲ TZ 20

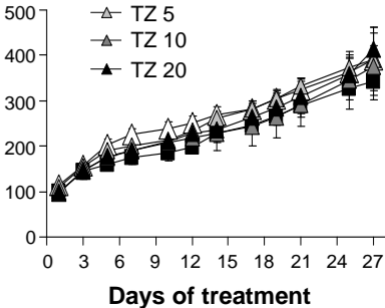

Supplement: Additional file 1 — Efficacy of TZ in JIMT-1 tumors. JIMT-1 xenografts (s.c.) were established in Rag2M mice and animals were treated with vehicle (saline) or 2.5, 5, 10 and 20 mg/kg trastuzumab (TZ) (n = 6 animals/treatment group). Treatment was initiated on day 21 and administered as intra-peritoneal injections (i.p.) twice weekly for 4 weeks. [file 1471-2407-11-420-S1.PDF]
